# Are There Differences in Androgen Receptor Expression in Invasive Breast Cancer in African (Tanzanian) Population in Comparison With the Caucasian (Italian) Population?

**DOI:** 10.3389/fendo.2018.00137

**Published:** 2018-03-29

**Authors:** Sara Bravaccini, Sara Ravaioli, Dino Amadori, Emanuela Scarpi, Maurizio Puccetti, Andrea Rocca, Maria Maddalena Tumedei, Nestory Masalu, Jackson Kahima, Akwilina Pangan, Lucas Faustine, Alberto Farolfi, Roberta Maltoni, Massimiliano Bonafè, Patrizia Serra, Giuseppe Bronte

**Affiliations:** ^1^Istituto Scientifico Romagnolo per lo Studio e la Cura dei Tumori (IRST) IRCCS, Meldola, Italy; ^2^Azienda Unità Sanitaria Locale (AUSL) Imola, Imola, Italy; ^3^Bugando Medical Center, Mwanza, Tanzania; ^4^Department of Experimental, Diagnostic and Specialty Medicine, Alma Mater Studiorum, University of Bologna, Bologna, Italy

**Keywords:** androgen receptor, breast cancer, African patients, Caucasian patients, tumor subtypes

## Abstract

**Purpose:**

Androgen receptor (AR) has been shown to have prognostic implication on breast cancer (BC). Data on the biological features of African BCs are poor. We decided for the first time to compare AR expression of Tanzanian and Italian BC patients.

**Patients and methods:**

Of the 69 consecutive patients seen at the Bugando Medical Center (Mwanza, Tanzania) from 2003 to 2010, who underwent resection of primary BC evaluable for estrogen receptor, progesterone receptor (PgR), and HER2 only 65 were evaluable for AR by immunohistochemistry. Histopathological assessment and biomolecular determinations were performed at the Cancer Institute of Romagna [Istituto Scientifico Romagnolo per lo studio e la cura dei tumori (IRST)—IRCCS, Meldola, Italy]. Caucasian BC patients were selected from an electronic database and matched (1:2 ratio) for year of diagnosis and age at diagnosis.

**Results:**

The median age of patients at diagnosis was 51 (range 29–83) years for Tanzanian and 53 (range 26–86) years for Italian patients. Tanzanian patients harbored tumors with lower AR expression than Italian patients according to the median percentage of immunopositive tumor cells (30% versus 80%, *p* < 0.0001) and staining intensity (*p* = 0.0003). The proportion of AR negative patients was likewise higher among Tanzanian patients as regards both ≥1% and ≥10% cutoffs. AR-positive BCs were higher in luminal A and B tumors and decreased in triple-negative (TN) and HER2-enriched tumors in Tanzanian population.

**Conclusion:**

AR loss could represent an unfavorable prognostic marker in the African population. The high frequency of TN tumors with high AR expression could open new perspectives of therapy for population in this low income country.

## Introduction

The mortality for breast cancer (BC) in Tanzania is the second cause of cancer death after cancer of the uterine cervix, and the major part of cancer being diagnosed very late at advanced stage (1).

Breast cancer incidence estimated age standardized rates in sub-Saharan Africa range from 15 to 53 per 100,000 women, so lower than Western countries (1). Furthermore, BC in Africa shows a trend to increase. Nevertheless, the cancer incidence and staging reported for sub-Saharan Africa might be impaired due to the absence of correct diagnosis, poor access to care, limitations in technical resources and infrastructure, and the low quality of tumor data systems in Africa compared with those in Western countries (2). The cancer related mortality rates tend to be higher among women in sub-Saharan Africa because tumors are more aggressive and diagnosis is delayed.

The prognosis and treatment of BC patients depend on numerous clinical, pathological, and biological factors. Their evaluation is important to classify BC into categories, that have different impact on treatment choice and prognosis [luminal A (ER+ and/or PR+, HER2−, low Ki67); luminal B (ER+ and/or PR+, HER2+/−, high Ki67); HER2-enriched (ER−, PR−, HER2+); and triple-negative (TN) (ER−, PR−, HER2−)]. The current treatment choice for BC in developed countries is influenced by numerous factors, including the risk of relapse, breast conservation, and impact on fertility. Surgery and radiotherapy play an important role for the treatment of early BC. Tamoxifen, with or without ovarian suppression, is the standard of care for women in premenopausal status with ER+ BC, while aromatase inhibitors are preferred for postmenopausal women. Luminal B and TN BCs are commonly treated with anthracyclines and taxanes, since chemotherapy lacks of a single standard of care. In addition to chemotherapy, for HER2-enriched BC patients, 1-year adjuvant treatment with Trastuzumab is recommended (3).

However, due to the poorer pathology skills and infrastructures in most sub-Saharan African countries, hormonal receptors, HER2, and Ki67 are not routinely assessed, and BC patients often undergo hormone treatment even though the receptor status is unknown (4).

Then, proper infrastructures and improved professional human skills and technical facilities are necessary to permit the biological and pathological BC characterization for each patient (4, 5).

Some authors have reported that African-American premenopausal women have a higher likelihood of developing BC with the “TN” phenotype (6).

Androgen receptor (AR) in BC is commonly expressed in the 60–80% of luminal tumors, 50–60% of HER2-enriched, and in the 20–40% of TN tumors (7, 8).

The proliferative index of the cell population is one of the most important information on invasive tumors, due to its prognostic role and its usefulness for decision making of adjuvant therapy. This index has been shown to be negatively related to AR expression in estrogen receptor (ER)-positive and HER2-negative BC (9). Several information is available on AR status for Caucasian BC patients but its prognostic significance in invasive tumors is still very much open to debate (7, 10). In a recent study, Cochrane and colleagues concluded that an AR/ER ratio ≥2 was an independent predictor of disease-free and cancer-specific survival (10). In particular, the authors suggested that a high AR/ER ratio may influence BC response by increasing the risk of tamoxifen failure (10). In previous studies, we observed the prognostic value for AR/ER ratio in patients with ductal carcinoma *in situ* (DCIS) of the breast treated with surgery alone (11) and in a population of DCIS patients treated with surgery and with radiotherapy. Unfortunately, only few data are available on the biomolecular characterization of Tanzanian breast tumors. To improve cancer control and care in Mwanza (Tanzania), we made an international project involving a non-profit association (Associazione Vittorio Tison) and the local hospital in Mwanza, Bugando Medical Center (BMC), together with the major local and national health authorities, to open a Medical Oncology Unit and Pathology Laboratory in the hospital. For this research project and since few data exist on the biological features of sub-Saharan Africa BC population, we carried out a study for the comparison of AR expression in case series of African (Tanzanian) and Caucasian (Italian) BCs.

In our previous work, we observed that BC in Tanzanian patients at time of diagnosis more frequently presented a higher histological grade (mainly grade 3), more advanced clinical stage (III or IV), ER negativity and higher proliferation index than those in Caucasian patients (4). We concluded that TN tumors were more present in Tanzanian women than in Caucasian population (4).

In this work, we want to compare AR expression in these two populations of BC patients in the different tumor subtypes. The availability of anti-AR compounds such as bicalutamide, enzalutamide, or apalutamide could open up new avenues for the treatment of AR-positive tumors (12–14).

## Patients and Methods

In this study, we compared the biological characteristics of Tanzanian and Italian BC patients matched (ratio 1:2) for date and age at diagnosis. The Medical Scientific Committee of IRST IRCCS, the Ethical Committees of Area Vasta Romagna (Italy) and BMC (Tanzania), and the National Institute for Medical Research (Tanzania) approved the study. The informed written consent from the participants was obtained.

### Case Series

A total of 69 consecutive patients who underwent biopsy or surgical resection of primary BC from 2003 to 2010 at the BMC (Mwanza, Tanzania) were enrolled in this study. Formalin-fixed paraffin-embedded tissues were analyzed for AR expression in the Biosciences Laboratory of the Cancer Institute of Romagna (IRST IRCCS) in Meldola, Italy.

Breast cancers from Caucasian patients were randomly extracted from an electronic database (Log80) of the Pathology Unit of Morgagni-Pierantoni Hospital (Forlì, Italy) and matched with Tanzanian patients for year of diagnosis and age at diagnosis (maximum difference of 2 years). The former stratification factor was chosen to avoid biological material alteration due to the long enrollment period, and the latter was chosen because age can affect the analysis of biomarkers in BC.

The clinical and pathological assessments of these cases were performed at the Oncology Unit and Pathology Laboratory of BMC, while hormone receptor status [ER and progesterone receptor (PgR)], HER2 expression, and proliferative activity (Ki67) assessments were previously done at the Biosciences Laboratory of IRST IRCCS. In this work, only AR expression was analyzed for the entire case series in our Institute.

### Immunohistochemistry

Four-micrometer sections of neutral buffered formalin-fixed, paraffin-embedded tissue were mounted on positive-charged slides (Bio Optica, Milan, Italy).

Androgen receptor determinations were performed according to European Quality Assurance guidelines.

Immunostaining for AR was performed using the Ventana Benchmark XT staining system (Ventana Medical Systems, Tucson, AZ, USA), with Optiview DAB Detection Kit (Ventana Medical Systems). AR (SP107 Cell Marque, Ventana Medical Systems) antibody was ready to use, prediluted by supplier, and sections were incubated for 16 min. The sections were automatically counterstained with hematoxylin II (Ventana Medical Systems).

Androgen receptor-positive samples were classified by using two different cutoffs ≥1% and ≥10% of nuclear immunopositive tumor cells in the nucleus on the total of the tumor cells. Staining intensity (i.e., 0 absent, 1+ weak, 2+ moderate, and 3+ strong) was also analyzed to calculate *H* score defined as the product of the percentage of the immunopositive tumor cells and the staining intensity.

Moreover, positive and negative breast tissues were used as intra- and inter-assay controls for AR expression. All samples were evaluated by two independent observers. A disagreement of more than 10% of positive cells was resolved by consensus after joint review using a multihead microscope.

### Statistical Analysis

All the data were summarized using descriptive statistics (counts and frequencies for categorical variables, median, and range for continuous variables).

The chi-square test or Fisher’s exact test for categorical variables and the Wilcoxon–Mann–Whitney test for continuous variables were performed to identify significant differences between the Italian and Tanzanian populations. Spearman’s rank correlation test (*r*_s_ coefficient) was used to investigate the relation between AR and Ki67 status. Statistical tests were two sided and were significant for *p* values < 0.05. Analyses were performed using SAS software version 9.4 (SAS Institute, Cary, NC, USA).

## Results

### Clinical Characteristics of Tanzanian and Italian BC Patients

199 patients were included in the study: 69 patients from Tanzania (100% African ethnicity) and 130 from Italy (100% Caucasian ethnicity). Of 69 Tanzanian BC cases of the overall series only 65 had tumor tissue to assess AR status and were matched with 130 Italian BC patients for age and date of diagnosis. The clinical–pathological features of the two case series are reported in Table 1. The median age of patients at diagnosis was 51 (range 29–83) years for African patients and 53 (range 26–86) years for Caucasian patients. Tumors from patients of the two populations were associated with a higher histological grade (mainly grade 3) even if the differences were not statistically significant (*p* = 0.258). Moreover, the invasive ductal cancer represented the 92.3% of the African BC population and the 81.5% of the Caucasian one. Tanzanian patients presented more frequently disease at advanced stage (III–IV) than Italian patients (*p* < 0.004) (Table 1). Luminal A tumors were 4 (6.1%) and 23 (19%), while luminal B tumors were 28 (43.1%) and 43 (35.6%) in the African and Caucasian population, respectively (Table 2). LB-HER2-enriched tumors were 20 (30.8%) and 34 (28.1%) and TN tumors were 13 (20%) and 5 (4.1%) in the African and Caucasian population, respectively (Table 2).

**Table 1:** Clinical and pathological features of the African and Caucasian population.

	African population	Caucasian population	
**Variable**	**Median (range)**	**Median (range)**	*****p*****

Age (years)	51 (29–83)	53 (26–86)	0.100

**Variable**	*****N*** (%)**	*****N*** (%)**	*****p*****

**Histological types**			
Invasive ductal carcinoma	60 (92.3)	106 (81.5)	0.085
Invasive lobular carcinoma	3 (4.6)	17 (13.1)	
Others	2 (3.1)	7 (5.4)	
**Histological grade**			
1	3 (4.6)	4 (3.1)	0.258
2	14 (21.5)	45 (34.6)	
3	34 (52.3)	56 (43.1)	
Unknown	14 (21.5)	25 (19.2)	
**Clinical stage**			
I	4 (6.2)	30 (23.1)	**0.004**
II	10 (15.4)	49 (37.7)	
III	12 (18.5)	17 (13.1)	
IV	12 (18.5)	23 (17.7)	
Unknown	27 (41.5)	11 (8.4)	

*The bold font is used for statistically significant values (p < 0.05)*.

**Table 2:** Distribution of tumor subtypes in African and Caucasian populations.

Tumor subtypes	African population*N* (%)	Caucasian population*N* (%)	*p*
LA	4 (6.1)	23 (19.0)	0.658
LB	28 (43.1)	43 (35.6)	
LB-HER2E	20 (30.8)	34 (28.1)	
TN	13 (20.0)	5 (4.1)	
HER2E	–	16 (13.2)	
Unknown/missing	–	9	

*LA, luminal A-like; LB, luminal B-like HER2-negative; LB-HER2E, luminal B-like HER2-positive; TN, triple-negative; HER2E, HER2-enriched non-luminal*.

### AR Biological Characteristics in the African and Caucasian Populations

**Figure 1:**
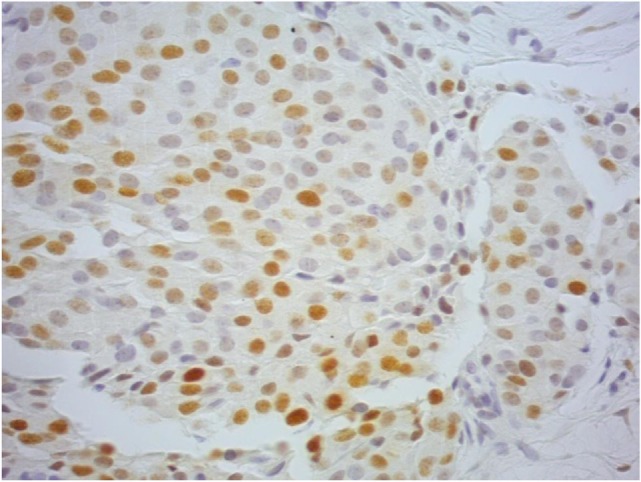
A Tanzanian ductal invasive carcinoma showing androgen receptor positivity in the nucleus of tumor cells, presenting different staining intensity (40× magnification). This case globally was evaluated as 2+.

The median AR expression (% of immunopositive tumor cells in the nucleus) in Tanzanian BC patients was 30 (range 0–100) and in Caucasian population was 80 (range 0–100) (*p* < 0.0001) (Figure 1). The median *H* score was 180 (range 10–300) in Tanzanian and 240 (0–300) in Caucasian patients (*p* = 0.109) (Table 3). AR staining intensity differed significantly between the two populations (*p* = 0.0003) (Table 3; Figure 1).

**Table 3:** Median values of androgen receptor (AR) %, *H* score, and staining intensity in African and Caucasian population.

	African population	Caucasian population	
		
	Median value (range)	Median value (range)	*p*
AR %	30 (0–100)	80 (0–100)	**<0.0001**
AR *H* score	180 (10–300)	240 (0–300)	0.109

**AR intensity**	*****N*** (%)**	*****N*** (%)**	*****p*****

0	22 (33.9)	22 (16.9)	**0.0003**
1+	4 (6.1)	0	
2+	13 (20.0)	20 (15.4)	
3+	26 (40.0)	88 (67.7)	

*The bold font is used for statistically significant values (p < 0.05)*.

Significant differences for AR expression between the two populations were observed with a great number of Tanzanian BC patients negative for AR expression both considering ≥1% and ≥10% as cutoff values (Table 4).

**Table 4:** Androgen receptor (AR) in Caucasian and African populations.

Cutoff	Results	African population*N* (%)	Caucasian population*N* (%)	*p*
AR ≥ 1%	Negative	22 (33.8)	22 (16.9)	
	Positive	43 (66.2)	108 (83.1)	**0.008**

AR ≥ 10%	Negative	25 (38.5)	27 (20.8)	
	Positive	40 (61.5)	103 (79.2)	**0.009**

*The bold font is used for statistically significant values (p < 0.05)*.

Androgen receptor positivity was more frequently observed in luminal A and B tumors than TN and HER2-enriched tumors in Tanzanian population (Table 5).

**Table 5:** Androgen receptor (AR) distribution in the different tumor subtypes of African and Caucasian populations.

	Primary tumor subtype					*p*
	LA*N* (%)	LB*N* (%)	LB-HER2E*N* (%)	TN*N* (%)	HER2E*N* (%)	
**African population**						
AR median value (range)	80 (10–100)	60 (0–100)	0 (0–90)	15 (0–90)	–	**<0.0001**
AR-negative (<1%)	0	1 (3.6)	15 (75.0)	6 (46.1)	0	
AR-positive (≥1%)	4 (100)	27 (96.4)	5 (25.0)	7 (53.9)	0	**0.0001**
AR-negative (<10%)	0	3 (10.7)	16 (80.0)	6 (46.1)	0	
AR-positive (≥10%)	4 (100)	25 (89.3)	4 (20.0)	7 (53.9)	0	**0.0005**
**Caucasian population**						
AR median value (range)	90 (0–100)	90 (0–100)	70 (0–100)	5 (0–90)	30 (0–80)	**<0.0001**
AR-negative (<1%)	3 (13.0)	6 (14.0)	6 (17.6)	2 (40.0)	5 (31.2)	
AR-positive (≥1%)	20 (87.0)	37 (86.0)	28 (82.4)	3 (60.0)	11 (68.8)	0.070
AR-negative (<10%)	4 (17.4)	6 (14.0)	7 (20.6)	3 (60.0)	7 (43.7)	
AR-positive (≥10%)	19 (82.6)	37 (86.0)	27 (79.4)	2 (40.0)	9 (56.3)	**0.010**

*LA, luminal A-like; LB, luminal B-like HER2-negative; LB-HER2E, luminal B-like HER2-positive; TN, triple-negative; HER2E, HER2-enriched non-luminal*.

*The bold font is used for statistically significant values (p < 0.05)*.

In addition, we evaluated the correlation between AR and Ki67 status in primary tumors. In the overall series of African and Caucasian tumors taken together, the *r*_s_ is −0.24 (*p* = 0.002), for the former the *r*_s_ is −0.21 (*p* = 0.209) and the latter showed an *r*_s_ of −0.23 with a *p*-value of 0.010.

## Discussion

Information on BC biomarkers is poor in the majority of low-resource countries, such as Sub-Saharan Africa. It is worthy of note that pathology capacity and infrastructures are insufficient in most parts of sub-Saharan Africa (4, 5, 15). In a previous work, we observed that the main problem in BC tissues from Tanzanian patients was the high percentage of not evaluable cases by immunohistochemistry, due to suboptimal fixation which compromised tissue morphology. The poor fixation exerts a great influence on the detection of biomarkers (4). HER2-positive, ER, and PgR negative, highly proliferating tumors were more frequently observed often in Tanzanian women than in Caucasian patients (4).

These highly aggressive biological patterns, with very advanced stage at diagnosis, could be considered the principal reasons for the high BC mortality rate in this African population. Then, the search for new biomarkers that could be used in the clinical practice is still an open issue and even more the identification of biomarkers to optimize the treatment choice.

Androgens have been thought to play an important role in BC. Even if we know that our results are preliminary due to low number of cases analyzed, in our knowledge this is the first study that compares the pathological and the biological features and AR expression in invasive BC in African (Tanzanian) and Caucasian (Italian) case series. Another study evaluated AR expression in Ghanaian BC patients. They found a lower percentage (24%) of AR-positive tumors (defined by ≥10% cutoff) among TN BCs (16).

Androgen receptor expression in Tanzanian BC patients was lower than the Caucasian population in terms of percentage, *H* score, and staining intensity. We are in agreement with Thike and colleagues that reported that the lower AR expression reflects the higher aggressiveness of tumors, but their study was performed in a different ethnicity, such as Asian population (17). AR is normally present also in normal tissue, and its presence is normally higher in well-differentiated cancers than undifferentiated tumors. The lower AR expression in African respect to Caucasian patients might be a consequence of a major tumor aggressiveness (low hormonal receptor expression and highly proliferating tumors) and probably of a different carcinogenesis (4).

Specifically, low AR levels have a scant transcriptional output, whereas they consistently activate extranuclear signaling pathways (i.e., Src tyrosine kinase, or PI3-K, or the filamin A-dependent pathway) leading to massive proliferation and invasiveness of target cells (18).

Moreover, an interplay between AR and ER has been known, and it is exerted at the level of estrogen responsive elements (19). The cross talk between AR and ER (alpha or beta) in human breast and prostate cancer cells has been known for long time. It occurs also at non-genomic levels.

Migliaccio and colleagues demonstrated that a non-genomic interplay between AR and ER can occur at protein level involving Src tyrosine kinase and epidermal growth factor receptor (20, 21).

Several coregulators balance the activity of these two hormone receptors and their interactions in different clinical settings. Some therapeutic approaches can be based on blocking this cross talk (22).

In Caucasian patients, AR was seen to be more expressed in luminal tumors than TN tumors, and its presence seems to be related to a better prognosis in ER-positive tumors (23–26). The AR expression for Tanzanian patients had the same trends to that observed in Caucasian population among the different tumor subtypes.

In the Tanzanian clinical practice, all patients underwent adjuvant hormonal therapy without testing the receptors. It means that only the fraction of ER-positive patients would benefit from this type of treatment. On the other hand, the majority of the patients (about 70%) were exposed to hormonal therapy unnecessarily, with subsequent side effects and additional costs in a low income country. The availability of new anti-AR compounds could lead to treat the patients with reduced recurrence, mortality, and costs (4, 5).

This area needs further investigation as the data on the differences of gene expression profiles in BC patients of various ethnicities are still controversial (27, 28). This study showed a tendency to a low expression of both hormonal receptors and AR in Tanzanian BCs. The significant proportion of AR-positive TN BCs could open new perspectives to treat these patients with anti-AR compounds.

The new availability of AR inhibitors such as bicalutamide, enzalutamide, and apalutamide approved for prostate cancer, suggests the possibility of their use also in AR-positive BC patients, even if AR seems to have different functions depending on BC subtypes (e.g., luminal or TN). It seems that in ER negative BCs AR expression does not have a clear prognostic effect (7), but it can predict response to AR inhibitors (29, 30).

It is recommended to introduce in Tanzania routine testing for these markers before initiation of hormonal therapy and also to consider an anti-AR therapeutic approach. Further analyses are ongoing to evaluate the role of other biomarkers in Tanzanian BCs.

## Ethics Statement

The study was approved by the Medical Scientific Committee of IRST IRCCS, the Ethical Committee of Area Vasta Romagna (Italy) and BMC (Tanzania), and the National Institute for Medical Research (Tanzania).

## Author Contributions

SB and DA designed the study. AR, NM, JK, AP, LF, AF and RM were responsible for patients’ recruitment. SR and MT performed the experiments. PS performed data management. ES performed the statistical analyses. MP and SB performed the immunohistochemical evaluation of all the samples. SB and GB interpreted the results and drafted the manuscript. MB revised the text. All the authors read and approved the present version of the manuscript for submission.

## Conflict of Interest Statement

The authors declare that the research was conducted in the absence of any commercial or financial relationships that could be construed as a potential conflict of interest.
